# Antimicrobials from Cnidarians. A New Perspective for Anti-Infective Therapy?

**DOI:** 10.3390/md14030048

**Published:** 2016-03-08

**Authors:** Gian Luigi Mariottini, Irwin Darren Grice

**Affiliations:** 1; 2 d.grice@griffith.edu.au

**Keywords:** Cnidaria, antibiotics, antimicrobials, drug discovery, anti-infective therapy

## Abstract

The ability of microbes to counter the scientific and therapeutic advancements achieved during the second half of the twentieth century to provide effective disease treatments is currently a significant challenge for researchers in biology and medicine. The discovery of antibiotics, and the subsequent development of synthetic antimicrobial compounds, altered our therapeutic approach towards infectious diseases, and improved the quality and length of life for humans and other organisms. The current alarming rise in cases of antibiotic-resistance has forced biomedical researchers to explore new ways to recognize and/or produce new antimicrobials or to find other approaches for existing therapeutics. Aquatic organisms are known to be a source of compounds having the potential to play a role in fighting the battle against pathogenic microbes. In this connection, cnidarians occupy a pre-eminent role. Over the past few decades several studies have explored the antimicrobial/antibiotic properties of cnidarian extracts with the aim of isolating compounds possessing useful therapeutic features. This paper aims to review the existing data on this subject, taking into account the possible utilization of identified compounds.

## 1. The Discovery of Antibiotics and the Antibiotic Era

The discovery and development of antibiotics and the consequent utilization of antibiotic therapy signaled a milestone in the history of biology and medicine. Antibiotic therapy allowed for the treatment of dangerous and fatal diseases, leading to a remarkable improvement in health conditions for humans, as well as increasing life expectancy. In addition, antibiotic therapy provided considerable health benefits for animals and vegetation, improving the production and utilization of reared organisms and human food crops.

Human exposure to antibiotics started reportedly in the ancient world [1]. Traces of tetracycline have been discovered in human skeletal remains from Nubia (Sudan) dating back to 350–550 CE [2], as indicated in mass spectrometry studies on bone extracts [3]. These reports on the occurrence of tetracycline in ancient bones suggest dietary exposure to tetracycline-containing materials. Furthermore, Cook *et al.* [4] report in a fluorochrome-labeling study of human skeletal samples from the Dakhleh Oasis (Egypt) dating back to the late Roman period indications of the presence of tetracycline in the diet. The apparent scarce incidence of infectious diseases in the Sudanese Nubian population [5] and the lacking of bone infections in samples coming from the Dakhleh Oasis [4] support this assumption.

The discovery and development of antibiotics started with the initial formulation of the “germ theory of disease” proposed by Louis Pasteur in the nineteenth century [6]. This theory set up the concept that microorganisms are responsible for infectious diseases. This proposal of Pasteur was confirmed later by Robert Koch, who demonstrated the microorganism-disease causal relationship. Interesting observations were made late in the 1800s by an Italian physician, Vincenzo Tiberio, who studied the “soluble principles” produced by moulds *Aspergillus flavescens*, *Mucor mucedo* and *Penicillum* (*Penicillium*) *glaucum*, observing bactericidal activity after experimental typhus and cholera infections [7]. These observations foreshadowed by over thirty years the official discovery of penicillin by Alexander Fleming. Unfortunately, these observations were not seriously considered by the scientific community and the early observations provided no significant contribution to the development of anti-infective therapy.

Subsequently, Paul Ehrlich suggested the concept that selective drugs can be utilized to fight definite microorganisms without damaging host cells. Further studies in 1930s led Gerhard Domagk to discover the antibacterial properties of Prontosil rubrum, which was demonstrated to be active against infections in laboratory animals, but inactive *in vitro*. Around this time infected patients were treated with Prontosil rubrum in France by Jacques and Therese Tréfoüel, who discovered patients excreted sulphanilamide, a compound with interesting antibacterial properties *in vitro* and *in vivo* [8].

Strictly though, the first “true” antibiotic to be discovered was Penicillin, after Fleming’s observation of the lack of colonies of bacteria in portions of a petri dish covered by moulds [9]. Clearly, as previously perceived [7], something produced by the mould prevented the bacteria from growing by inhibiting the activity on the bacterial cell wall [9].

The discovery of Fleming had a real world application, but only after further experimental work by Florey, Chain and Heatley, who characterized penicillin and observed the inhibition of *Streptococcus pyogenes* in infected mice. The discovery of penicillin enabled several life threatening bacterial infections to now become treatable, and started the “antibiotic era” accompanied by the industrial production of antibiotics [6]. Subsequently, several other significant antibacterials, such as streptomycin, the first efficient remedy against tuberculosis [10], and cephalosporins were discovered or synthesized. Cephalosporin (Cephalosporin C) was first identified by the Italian scientist Giuseppe Brotzu studying the fungi *Cephalosporium acremonium* in sewer-contaminated waters near Cagliari harbour [11,12,13], and subsequently isolated [14,15,16,17].

Some of the early antibiotics are now known to be unacceptably toxic and are therefore not used in antimicrobial therapy. Some however, such as adriamycin, bleomycin and mitomycin are presently used as cytostatic/cytotoxic agents in cancer therapy [6].

## 2. Antibiotic/Antimicrobial Resistance

Bacteria belong to a biological world different from that of eukaryotes, where their growth and survival can be affected by agents ineffective against animal or vegetal cells [6]. In spite of this, microbes have unfortunately developed genetic modifications and mobilized molecular defense mechanisms that are able to protect them against antibiotics [6].

The understanding of microorganism resistance implies the distinction between “antibiotics” and “antimicrobials”. Waksman defined an antibiotic as “*a chemical substance, produced by micro-organisms, which has the capacity to inhibit the growth of and even to destroy bacteria and other micro-organisms*” [18,19]. According to the Michigan State University website [20], an antibiotic is “*a low molecular substance produced by a microorganism that at a low concentration inhibits or kills other microorganisms*”, while “*an antimicrobial is any substance of natural, semisynthetic or synthetic origin that kills or inhibits the growth of microorganisms but causes little or no damage to the host*”. In the same website we can read that “*all antibiotics are antimicrobials, but not all antimicrobials are antibiotics*”. At present, “antibiotic” is often considered to be a synonym for “antibacterial” [19].

Similarly, antibiotic- and an antimicrobial-resistance exists [21]. The first definition refers to the resistance demonstrated by pathogenic bacteria, and the second one refers to the resistance demonstrated by all “microbes”, such as bacteria, viruses, fungi and also by parasites. According to the World Health Organization (WHO) [21], antimicrobial resistance is the “*resistance of a microorganism to an antimicrobial drug that was originally effective for treatment of infections caused by it*”. The resistance can be observed in infections by viruses, bacteria, fungi, and parasites, and is basically due to the misuse of antimicrobial drugs.

Antibiotic resistance can develop in a short time owing to the short replication time of bacteria, a factor which allows them to evolve fast in comparison to other organisms; it can also be due to genetic manipulations performed by bacteria, with a consequent fast adaptation [6]. The resistance to antibiotics implies the transfer of DNA between bacteria, which implies, as a consequence, the “contagious resistance”, namely the infection of antibiotic-susceptible bacteria with resistance genes, resulting in a contagious multi-resistance [6].

Antimicrobial resistance is a significant threat to human society, whereby new resistance mechanisms are emerging globally [21]. For example, in the last few years an increase in resistance to HIV drugs have been detected, as well as reports of a remarkable widespread increase in new cases of multidrug-resistant tuberculosis and malaria [21].

Multidrug resistance develops due to chromosomal mutations of bacteria, along with the acquisition of extra-chromosomal elements, such as mobile DNA segments (plasmids, transposons, and integrons), from the environment [22,23].

According to WHO [21] antimicrobial resistance is supported by some fundamental factors, such as “*poor infection control practices, inadequate sanitary conditions and inappropriate food-handling*”, which lead to increased risk of prolonged illness, worsening of outcomes and, in some cases, death.

WHO [21] also states that resistance occurs mainly in sites where the use of antimicrobials has been (and is) more massive (e.g., hospitals). An example is the occurrence of hospital-acquired infections by methicillin-resistant *Staphylococcus aureus* (MRSA) causing 64% higher death rates than non-resistant strains or multidrug-resistant Gram-negative bacteria. Another aspect worthy of consideration is the effect of long term infectious conditions, thus increasing the time for resistant infections to occur. In addition, the costs and the resources involved in the care of patients infected with resistant microorganisms are very high.

The outcome of these situations is that several diseases which were practically eradicated, or where the therapy was routine, such as for some urinary infections, gonorrhoea, intestinal infections, tuberculosis and malaria are again threatening humanity. Resistance to antiretroviral therapy in HIV treatment is a current problem adding to the normal therapy for this disease. In fact, all anti-HIV therapies have evolved-resistance, due to the very high mutation rates of the virus.

Another aspect worthy of consideration is the capability of microbes to grow in the biofilm mode. Bacteria are known to colonise surfaces, growing as multicellular biofilm structured communities, which are surrounded by a self-produced polymeric matrix in which they are included. This feature of microbial communities allow them to survive in adverse conditions and makes a barrier against host defences, antibodies and drugs, causing persistent and chronic infections. Recent data estimates that nearly 65% of infections are due to the biofilm mode of growth [24,25]. The research on this lifestyle of microbes includes genome/proteome analyses and testing of antibiotic susceptibility [24].

Proteomic methodologies have contributed to our current understanding of antimicrobial resistance mechanisms [26]. Several studies have shown that resistant microbial strains have an increased abundance of some proteins involved in virulence, antibiotic resistance, and DNA protection, as well as peculiar outer membrane proteins, in comparison with wild-type strains [27]. Conversely, other proteins, such as those involved in glycolysis, amino acid transport, synthesis and protein transport, cell division, oxidative stress response, and energy metabolism have been observed to decrease in resistant strains [27]. Virulence and antibiotic resistance are reportedly associated with specific gene clusters, which have been named “pathogenicity islands”, which are passed to other bacteria by gene transfer [28,29,30].

In general, the role of “omics” approaches, integrating genomics, proteomics and transcriptomics are thought to be fundamental in the research for antibiotic/antimicrobial resistance and in the management of microbial resistance [31]. Notably, proteomics allows one to identify and characterize the proteins from a proteome with the production of useful information, to analyze the expression of proteins and drug targets associated to a disease, and to describe protein interactions, with the final purpose to determine the mechanisms of resistance [32].

## 3. The Role of Natural Compounds in Drug Discovery

Many drugs utilized for modern therapy, mainly in oncology and in infectious diseases, are known to be derived from natural sources [33]. Furthermore, many synthetic drugs are based on the chemical structure of natural compounds. For this reason, organisms such as microbes, plants, and animals are considered interesting sources of useful substances with pharmacological properties [34]. Of particular interest here are aquatic organisms that have been little explored as sources of bioactive molecules for the development of novel chemotherapeutics [35,36]. Factors such as the difficulty in collecting specimens, the rarity and quantity of active extracts and the wide heterogeneity of compounds have been enumerated to explain the current difficulties encountered to develop new therapeutic agents from aquatic organisms [37].

Natural venoms are included among the sources of compounds of pharmacological interest (including antimicrobial) [38]. Snake venoms are multifunctional mixtures, known to have anti-bacterial, anti-viral and anti-parasitic properties [39,40]. Other organisms are also known to contain constituents possess antimicrobial properties [41,42,43].

## 4. Cnidarians as a New Option for the Development of Drugs?

Cnidarians are proposed as a promising source of compounds useful as new drugs [44,45]. Interestingly, these organisms occupy a peculiar position among venomous animals, being sometimes at the top of the food chain and being able, in the instance of wide-outbreaks they have displaced all greater organisms, monopolizing their environmental resources, food inputs and the available space [46,47,48,49].

The physiology and ecology of cnidarians is closely linked to their venomous properties. Cnidarian venoms are synthesized in tissues. Presumptively they are then transferred into the nematocysts [50], which are double-walled intracellular capsules produced by the Golgi apparatus of specialized cells, the nematocytes [51]. The nematocyst is filled with a venomous fluid and includes a tightly spiralized filament that is able to facilitate injecting the capsular contents into the prey [44]. Cnidarians are included among the most venomous organisms and owing to their frequent outbreaks [49,52,53,54], they represent an emergent problem at a global level. As a matter of fact, even though many cnidarians induce only local symptoms, several of them, notably some Australian species, can be life-threatening to humans. On the whole, cnidarian venomousness seem to be due mainly to the proteins included in venoms [55], and to the phospholipase activity which causes damage (pore formation, oxidative stress, alteration of ion exchange, alteration of membrane permeability) to compromised cells [56,57,58].

The potential of cnidarians to be utilized as a drug source is demonstrated by the remarkable number of active molecules (over 2000) isolated from these organisms between 2000 and 2010 [45]. Some of these compounds have been used in traditional medicines to treat hypertension, respiratory, genitourinary, dermatological, neurological, and haematological diseases [59,60,61,62]. To date however, none of the identified compounds extracted from cnidarians have been effectively developed as drugs. Although, Pseudopterosin extracted from the sea whip *Pseudopterogorgia elisabethae* [63], Eleutherobin from *Eleutherobia* sp. (soft coral) and *Erythropodium caribaeorum*, and Sarcodictyns from some corals have been investigated in preclinical studies [64].

Notably, cnidarians lack of a traditional protection system present in upper-level organisms, such as impermeable barriers (e.g., cuticle, exoskeleton), hemolymph, migratory phagocytic cells. Additionally, they live in a habitat that is normally full of viruses, bacteria, protists and parasites to which they are constantly exposed and yet they do not seem vulnerable to pathogens [65]. Therefore, their defense systems are of particular interest, in that they likely contain compounds useful to counteract these microbes.

In this connection, this paper aims to review the existing scientific literature on this subject, considering the data pertaining to the different classes of cnidarians, and taking into account the possible future utilization of compounds extracted from these organisms. A separate section has been devoted to the antiviral effects and in conclusion a section pertaining to the anti-parasitic properties of cnidarian extracts has been included.

## 5. Antimicrobials from Cnidaria

### 5.1. Freshwater Cnidaria

#### Antimicrobials from Hydrozoa

The release of antimicrobials and anti-proteinases by epithelial cells in response to external antigens as evidenced by an increased expression of encoding genes was observed in *Hydra* [65]. In a recent paper, Bosch *et al.* [66] stated that the freshwater hydrozoans *Hydra magnipapillata* possess potent antimicrobial peptides in ectodermal and endodermal epithelia, which compensate for the lack of phagocytic cells and protective layers. These peptides can counteract the environmental pathogens and prevent the entry of infectious microbes into the body. This was perceived as an epithelium-mediated innate immune response based on unconventional Toll Like Receptor (TLR) signaling. *Pseudomonas aeruginosa* growing on *Hydra* surfaces were seen to induce changes of ectodermal cell morphology which become rounded, form blebs at the surface, and produce granules resembling those of immune cells typical of higher organisms. Defense mechanisms were shown also in the endoderm. In extracts from *Hydra,* Hydramacin-1, a peptide with strong microbiocidal activity, was isolated. Hydramacin-1 seems to be inducible by microbial products, such as lipopolysaccharide (LPS), in a dose-dependent manner [66]. In addition, Hydramacin-1 remarkably affected the growth of *Bacillus megaterium*, certain resistant *Escherichia coli*, *Klebsiella oxytoca* and *Klebsiella pneumoniae* strains, but exhibited weak activity against *Pseudomonas aeruginosa*, some important Gram-positive pathogens and fungi [66]. The gene Periculin-1 expressing the Periculin-1 peptide was found in endodermal epithelial cells and in ectodermal interstitial cells of *Hydra*. This peptide was shown to be involved in *Hydra* host defense and in antimicrobial activity against *Bacillus megaterium* (LD_90_ = 0.2–0.4 μM) [66]. The structure and amino acid sequence of Hydramacin-1 was subsequently studied and the antimicrobial activity present against Gram-negative bacteria, including multiresistant strains [67].

Augustin *et al.* [68] identified multiple short peptides (arminins) in *Hydra magnipapillata* consisting of a highly conserved *N*-terminal region with negative charge, and of a scarcely conserved positively charged C-terminal region. The peptides showed broad-spectrum activity against human pathogens. The recombinant c-arminin 1a produced in *Escherichia coli* was shown to have antibacterial activity, affecting the growth of *Escherichia coli* DH5a, *Bacillus megaterium* ATCC 14581 and *Staphylococcus aureus* ATCC 12600, at Minimal Bactericidal Concentrations (MBC) 0.2, 0.1, and 0.4 μM, respectively. Further antibacterial activity was seen against methicillin-resistant *Staphylococcus aureus* (MRSA) strains at MBC 0.4–0.8 μM, with LD_90_ of 0.2 μM for all strains. Ultra-structural evaluations emphasized the disruption of cell walls of bacteria induced by arminin1. Arminin did not lose antibacterial activity under the conditions occurring in human blood and did not show adverse effects to human erythrocytes. These properties make arminin a very interesting antibacterial agent for the treatment of infections due to resistant microbes [68].

Gland cells from *Hydra magnipapillata* strain 105 reportedly produce a serine protease inhibitor, kazal2, possessing bactericidal activity against *Staphylococcus aureus* ATCC12600, resulting in a MIC of 0.7–0.8 μM for native kazal2, in a MIC ranging from 33 to 38 μM for recombinant kazal2 domains 1–3, and in a MBC of 33–38 μM at pH 7.4. The use of kazal2 as anti-staphylococcal agent has been suggested [69]. Furthermore, as kazal2 did not affect the growth of *E. coli*, Augustin *et al.* [69] proposed that it acts by inhibiting a protease essential for *S. aureus*, but not essential for *E. coli*.

Subsequently, Franzenburg *et al.* [70] studied gene expression leading to the production of antimicrobial peptides of the arminin family in *Hydra*. He reports that the antimicrobial peptides are highly expressed and their production profiles are species specific. *Hydra vulgaris* and *Hydra magnipapillata* showed similar arminin expression, although higher levels of expression were evidenced in *Hydra vulgaris* whose endodermal epithelial cells constituted the exclusive site of expression.

Kasahara and Bosch [71] observed a strong correlation between the amount of neurons in tissues of *Hydra magnipapillata* and the activity against *Bacillus subtilis* strain DSM 347 and *E. coli* strain XL blue. Nerve-free tissues of *Hydra magnipapillata* sf-1 mutants obtained by exposing polyps to 25 °C (non-permissive temperature) showed higher activity than normal tissues against *E. coli*, with more than three times the effect after eight days, and more than four times the effect after 12 days, and a lessened, but still evident increase of activity against *B. subtilis*. Therefore, the amount of neurons could influence the innate immune response as well as the antimicrobial activity in *Hydra*.

The antimicrobials isolated from freshwater cnidarians, along with their activity is reported in Table 1.

### 5.2. Marine Cnidaria

#### 5.2.1. Antimicrobials from Anthozoa

During the early 1990s, the activity of polar and non-polar extracts from soft corals (Anthozoa: Octocorallia) *Plexaura homomalla*, *Pseudoplexaura flagellosa*, *Plexaurella fusifera*, *Eunicea clavigera*, *Eunicea tourneforti*, *Eunicea laciniata*, *Eunicea calyculata* (Plexauridae), and *Pseudopterogorgia americana* (Gorgonidae) were tested on marine bacteria and on human pathogens (*Vibrio harveyi*, *P. aeruginosa*, *S. marcescens*, *S. aureus*, *B. megaterium* and *E. coli*). The inhibition of microbes seeded in agar plates was evaluated after application of 6.2 mm paper disks soaked in cnidarian extracts and incubated overnight at 37 °C. The measured inhibition zones were converted to activity scores (1 for each 0.5 mm of inhibition zone; 0.5 for less, but visible inhibition) which were added to obtain the final result. *P. homomalla* and *P. flagellosa* extracts exhibited the main antimicrobial activity, with inhibition scores of 18.3 and 15.0, respectively [72].

Subsequently, the structure of the active compounds isolated from the West Indian gorgonian coral *Pseudopterogorgia elisabethae* were determined using NMR spectroscopy experiments, with the compounds found to contain an uncommon benzoxazole moiety. The activity of two isolated diterpenoid alkaloids, namely pseudopteroxazole and seco-pseudopteroxazole were assessed on *Mycobacterium tuberculosis* H37Rv, where strong growth inhibition was observed as induced by pseudopteroxazole (97% inhibition induced at 12.5 μg/mL) and a moderate/strong inhibition induced by seco-pseudopteroxazole (66% inhibition induced at 12.5 μg/mL) [73]. Subsequently, a minor constituent of the extract from *Pseudopterogorgia elisabethae*, homopseudopteroxazole (an alkaloid), was found to affect the growth of *M. tuberculosis* H37Rv, inducing an 80% growth decrease with a MIC value of 12.5 μg/mL [74]. Recently, interesting anti-tubercular properties were reported for pseudopterosins, pseudopterosin congeners, seco-pseudopterosins, synthesized pseudopteroxazoles and pseudopteroquinoxalines, four of these compounds were reported as being active against *M. tuberculosis* H37Rv with MIC values ranging from 13 to 15 μg/mL [75]. Notably, 21-((1H-imidazol-5-yl)methyl)-pseudopteroxazole, a semi-synthetic compound, showed strong activity against replicating and non-replicating persistent *M. tuberculosis*, and pseudopteroxazole was particularly active against resistant *M. tuberculosis* H37Rv strains [75]. A pseudopteroxazole derivative affected the growth of *Mycobacterium smegmatis* and *M. diernhoferi* (MIC values = 4 and 2 μg/mL, respectively) as well as that of Gram-positive MRSA and vancomycin-resistant *Enterococcus faecium* (IC_50_s = 3 and 7.5 μg/mL, respectively).

Seven pseudopterosins (PsG, PsP, PsQ, PsS, PsT, PsU, 3-O-Ac-PsU), two seco-pseudopterosins (seco-PsJ, seco-PsK), and the inter-converting mixture of non-glycosylated diterpenes (IMNGD) were reported as active against *S. aureus* (mean IC_50_ values of 2.33 μM for IMNGD, ranging from 2.97 to 20.23 μM for pseudopterosins, and from 4.20 to 6.52 μM for seco-pseudopterosins), and *Enterococcus faecalis* (mean IC_50_ values of 3.47 μM for IMNGD, ranging from 3.14 to 37.35 μM for pseudopterosins, and from 3.82 to 4.08 μM for seco-pseudopterosins), but were inactive against *Pseudomonas aeruginosa* and *Candida albicans* [76]. Inhibition of *S. aureus* was obtained with PsU, PsQ, PsS, seco-PsK and PsG with IC_50_ values of 2.9–4.5 μM. *E. faecalis* was inhibited by PsG, PsU and seco-PsK with IC_50_s ranging from 3.1 to 3.8 μM [76]. The above results have been compared with data obtained from the evaluation of other pseudopterosins (PsA-E, PsK, PsX and PsY) which were reported to give MIC values ranging from 4.2 to 8.8 μM [76]. Ata *et al.* [77] isolated four new diterpenes, elisabethin E, elisabethin F, pseudopterosin P, and pseudopterosin Q from the MeOH extract of *Pseudopterogorgia elisabethae*. Pseudopterosins P and Q exhibited selective activity against *Streptococcus pyogenes* (MICs: 0.8 and 1.0 μg/mL, respectively), *Staphylococcus aureus* (MICs: 2.0 and 2.3 μg/mL, respectively), and *Enterococcus faecalis* (MICs: 3.5 and 3.6 μg/mL, respectively).

During the last decade the first reports on the antimicrobial activity of crude aqueous methanol extracts from *Leptogorgia virgulata* were published [78]. The extracts inhibited the growth of *Vibrio harveyii* and *Micrococcus luteus* (extraction of >0.5 g of tissue), and of *E. coli* (extraction of 2.0 g of tissue). Subsequently, Tadesse *et al.* [79] tested the extracts from *Alcyonum digitatum* on Gram-negative and Gram-positive bacteria. *E. coli* and the fish pathogen *Listonella anguillarum* serotype O2 (Gram-negatives) exhibited resistance, while the Gram-positives *S. aureus* and *Corynebacterium glutamicum* were sensitive (MICs: 80 μg/mL). The antimycotic activity against *C. albicans* and *S. cerevisiae* was also assessed, observing sensitivity of *S. cerevisiae* (MIC: 160 μg/mL) [78].

In 2012 Chen *et al.* reported that fifteen guaiazulene-based terpenoids (anthogorgienes A–O) and eight analogues that were isolated [80] from the lipophilic extract of *Anthogorgia* sp. and tested on *S. aureus* and *Streptococcus pneumoniae* and on three fungi (*Aspergillus fumigatus*, *Aspergillus flavus*, *Fusarium oxysporum*). Moderate antimicrobial activity was exerted by three compounds: 1,7-guaiazulenequinone induced growth inhibition of *A. fumigatus*, *A. flavus*, *F. oxysporum*, *S. aureus*, and *S. pneumoniae* (IC_50_s of 13.34 μg/mL, 18.68 μg/mL, 15.04 μg/mL, 12.30 μg/mL, and 15.66 μg/mL, respectively). The aldehydic analogue was active against *A. fumigatus*, *A. flavus*, and *F. oxysporum* (IC_50_s of 19.50 μg/mL, 37.90 μg/mL, and 35.50 μg/mL, respectively), while anthogorgiene G induced selective inhibition of *S. aureus* and *S. pneumoniae* (IC_50_s of 18.03 μg/mL, and 12.67 μg/mL, respectively).

Li *et al.* [81] isolated six briarane diterpenoids (gemmacolides T–Y) and two analogs (juncenolide J and praelolide) from the sea-whip *Dichotella gemmacea* and reported the occurrence of weak antimicrobial properties of all compounds against the growth of *E. coli* (inhibition zone of 11.0–34.0 mm diameter). Treatment of fungi *M. violaceum* and *S. tritici* resulted in inhibition zones of 9.5–15.0 mm and 9.5–17.0 mm diameter, respectively.

Ethanol extracts from octocorals *Pacifigorgia media*, and *Pacifigorgia* sp. sampled from Baja California Sur (México) were found to possess antimicrobial activity against *Mycobacterium tuberculosis* H37Rv (96%–99% inhibition after treatment with 300 μg/mL, and 83%–98% inhibition after treatment with 100 μg/mL, using the BACTEC 460 system). *Pacifigorgia media* extract resulted in activity against *Mycobacterium avium* (80% inhibition after treatment with 300 μg/mL, using the BACTEC 460 system) [82].

As reported in two papers (Iguchi *et al.*, 1989; Liu *et al.*, 1992) cited by El Sayed *et al.* [83], anti-tubercular activity was shown by three steroids (litosterol, nephalsterol B, and nephalsterol C) extracted from *Nephthea* sp. Notably, litosterol and nephalsterol C were able to inhibit the growth of *M. tuberculosis* (90% and 96%, respectively), resulting in MIC values of 3.13 and 12.5 μg/mL, respectively. Other compounds (Calyculones A, B, C and H) were isolated from *Eunicea* sp. and tested against *M. tuberculosis* inducing 23%–43% inhibition at concentration of 6.25 μg/mL [84].

Three diterpenoids (gyrosanol A, B, and C) were isolated from *Sinularia gyrosa* collected at Dongsha Atoll off Taiwan; two of them (gyrosanol A and B) showed antiviral properties but were ineffective against *Enterobacter aerogenes*, *Salmonella enteritidis*, *Serratia marcescens*, *Shigella sonnei*, and *Yersnia enterocolitica* at concentration of 100 μg/disk [85]. Subsequently, two glycosides isolated from *Sinularia humilis* (sinularoside A and B) were shown to possess anti-mycotic and antibacterial properties comparable to ketoconazole and streptomycin, radii of the zones of inhibition ranging from 14 to 17 mm. These compounds exhibited activity against *Microbotryum violaceum*, *Septoria tritici* (fungi), and *Bacillus megaterium* (Gram-positive), but were ineffective against *E. coli* [86].

Moderate antimicrobial activity against *E. aerogenes*, *S. marcescens*, *Y. enterocolitica*, and *S. sonnei* was shown by erectathiol, a sesquiterpene isolated from *Nephthea erecta* at concentration of 166 μg/disk. Interestingly, this compound exhibited remarkable activity against *S. enteritidis* being more effective than ampicillin [87].

The alcohol extract of the Red Sea soft coral *Sarcophyton glaucum* yielded sarcophytolide, a lactone cembrane diterpene, which showed activity against *S. aureus* (Gram-positive) with MIC of 0.19 μg/mL, *P. aeruginosa* (Gram-negative) with MIC of 0.22 μg/mL, and *Saccharomyces cerevisiae* (yeast) with MIC of 0.13 μg/mL, but did not affect the growth of *E. coli* [88]. Among eleven compounds (sartrolides A–G, bissartrolide, and three analogues) isolated from *Sarcophyton trocheliophorum* only one (cembranolide) showed antimicrobial activity against *S. aureus* after 24 h of treatment (paper disk diffusion) with a MIC value of 125 μg/mL [89].

A comparison between the antimicrobial properties of extracts from Red Sea soft corals (alcyonaceans) *Litophyton arboreum*, *Rythisma fulvum*, *Heteroxenia fuscescens*, *Sarcophyton glaucum*, *Dendronephthya hemprichi*, *Xenia macrospiculata*, and stony (scleractinian) corals, *Acropora variabilis*, *Fungia scutaria*, *Fungia granulosa*, *Turbinaria* sp., *Stylophora pistillata*, and *Favia favus*, showed that the majority of soft corals (83%) affected remarkably the growth of the marine bacteria *Arthrobacter* sp. (two strains), and scarcely the growth of *Vibrio* sp., while stony corals showed very little or no activity [90]. The strongest antimicrobial properties against two strains of *Arthrobacter* were shown by *Xenia macrospiculata* extract at a mean concentration 41.9 mg/mL (mean inhibition zone of 4.6 and 9.7 mm). *Xenia macrospiculata* yielded a range of antimicrobial compounds among which desoxyhavannahine showed interesting activity (MIC = 48 μg/mL) [90].

With regard to hexacorals, during the late 1990s the defence mechanisms of 100 scleractinian corals were studied by Koh [91]. She tested the activity of extracts against marine bacteria and human pathogens (*Alteromonas rubra*, *Photobacterium damsela*, *Vibrio alginolyticus*, *Vibrio parahaemolyticus*, *V. harveyi*, *S. aureus*). Low sensitivity of *S. aureus* (inhibition zone of 1.8 ± 0.2 mm diameter) was observed. Subsequently, the activity of eggs from *Acropora elseyi*, *A. tenuis*, *A. hyacinthus*, *A. valida*, *A. cerealis*, *A. millepora*, *Echinopora lamellina*, *Goniastrea favulus*, *Favia pallida*, *Lobophyllia pachysepta* and *Montipora digitata* on the attachment and growth of bacteria was reported; the eggs of *Montipora digitata* induced growth inhibition of *V. harveyii* and *B. subtilis* [92].

Recently, thermo-stable proteases and antimicrobial peptides have been purified from the body and tentacles of the sea anemones *Actinia equina* and *Anemonia sulcata* and have had application for bio-cleaning and as antifungals [93]. The mucus of *A. equina* has been recently indicated as a source of antimicrobial lysozyme-like compounds, showing lysozyme-like activity and thereby possessing activity as a antifouling agent, countering the settlement of bacteria, as the primary colonizers in marine waters [94]. The activity of the mucus was found to be dependent on pH (maximum diameter of lysis was observed at pH 6.0), ionic strength, and temperature. Lysis was reported to increase after dialysis of mucus at pH 6.0, 0.175 ionic strength, and 37 °C temperature, resulting in a diameter of lysis of 16.2 ± 0.5 mm, corresponding to 2.21 mg/mL of hen egg-white lysozyme [94]. Considering that seawater is at a higher pH and ionic strength, the activity of this mucus against marine bacteria remains to be elucidated. The activity against *Micrococcus lysodeikticus* known to be useful for the assay of lysozyme, and the satisfactory results obtained at 37 °C make *A. equina* mucus an interesting perspect for future developments in fighting pathogenic microbes.

Regarding the anthomedusae, Fredrick and Ravichandran [95] assessed the antimicrobial properties of *Porpita porpita* crude extract on bacteria and fungi, reporting preliminary results on the activity against *K. pneumoniae* and *A. niger* (16 mm and 13 mm inhibition zone, respectively). Although quantification of the active amount/weight of extract was not possible.

Recently, colonies of marine bacteria *Bacillus* and *Pseudomonas* associated with *Sarcophyton glaucum* specimens rinsed with sterile seawater and subsequently homogenized, were spotted onto the surface of agar plates in which indicator pathogenic strains were seeded. Colonies were found to possess strong antimicrobial properties against *Staphylococcus aureus*, *Klebsiella pneumoniae*, *Pseudomonas aeruginosa*, *Vibrio fluvialis*, and fungi (*Penicillium* sp., *Aspergillus niger*, *Candida albicans*). This activity has been interpreted as a host defence mechanism against marine pathogens [96]. A similar host protection activity was supposed to be due to fungi isolated from the zoanthid *Palythoa haddoni*. The organic extracts of mycelia and the fermentation broth (1 mg/mL) of 49 fungi obtained from the homogenate of rinsed *P. haddoni* specimens, after removal of sediments and loosely attached microorganisms, were evaluated for antibacterial activity, showing that nearly 60% of the isolates (notably, two strains of *Cladosporium*, two strains of *Nigrospora*, and one strain of *Fusarium*) induced selective growth inhibition of *Nocardia brasiliensis*, *Vibrio parahaemolyticus*, and *E. coli*, with similar (or stronger) activity in comparison with ciprofloxacin. Two strains of *Cochliobolus*, and three strains of *Exserohilum* were active against *S. aureus*, *Tetragenococcus halophilus*, *E. coli*, *N. brasiliensis*, and *V. parahaemolyticus*. Five strains of *Nigrospora* inhibited selectively the growth of *S. epidermidis* [97].

The activity of antimicrobials from Anthozoa is reported in Table 2.

#### 5.2.2. Antimicrobials from Scyphozoa and Cubozoa

The antimicrobial properties of scyphozoans are less known. Ovchinnikova *et al.* [98] purified aurelin, a 40-residue antimicrobial peptide, from the mesoglea of *Aurelia aurita*. The peptide, having a molecular mass of 4296.95 Da was obtained through preparative gel electrophoresis and RP-HPLC. It was shown to inhibit the growth of Gram-positive (*Listeria monocytogenes*), and Gram-negative (*E. coli*) bacteria, with resulting MICs of 5.27 μM and 1.78 μM, respectively. The complete sequence of the peptide was determined and the similarity with defensins and K+ channel-blockers of sea anemones was emphasized, but no structural homology with other antimicrobial peptides was recognized. Subsequent research tested aurelin on *Bacillus megaterium*, *Bacillus mycoides*, *Bacillus subtilis*, *Micrococcus luteus*, *Micrococcus phlei*, *Staphylococcus aureus*, *Escherichia coli*, and *Pseudomonas aeruginosa* showing that this peptide is active only at rather high inhibitory concentrations. The MICs for *Bacillus megaterium* (strain B-392), and *Micrococcus luteus* (strain Ac-2229) were reported as 10 and 40 mM, respectively). [99]. The activity of aurelin is reported in Table 2.

Subsequently, Grant *et al.* [100] studied two fractions (<500 Da and >500 Da) derived from the extract of *Aurelia aurita*, observing that the <500 Da fraction was active mainly on *P. aeruginosa*, *Enterobacter aerogenes*, *Serratia marcescens*, and *S. aureus* with bacterial mortality of 34.7%, 33.6%, 31.6%, and 28.3%, respectively, after 10 h treatment. The fraction >500 Da was less effective, inducing a maximum bacterial cell death of 13.4% against *Micrococcus luteus*.

Recent data showed that the fractionated n-butanol extracts of venom of *Chrysaora quinquecirrha*, induced low and barely measurable damage against *S. paratyphi* (8.0 mm inhibition zone) and *A. niger* (9.0 mm inhibition zone) [101].

Regarding cubozoans, Morales-Landa *et al.* [102] tested the activity of crude extracts from *Carybdea marsupialis* (Carybdeida) against bacteria *Streptococcus faecalis*, *B. subtilis*, *M. luteus*, *E. coli*, *K. pneumoniae*, *Serratia marcescens*, *S. typhi*, and fungi *C. albicans*; 10 μg/mL did not produce any damage to microorganisms.

### 5.3. Antiviral Activity of Cnidarian Extracts

All available data on the antiviral activity of cnidarian extracts is recent. Gyrosanol A and B, two compounds isolated from *Sinularia gyrosa*, were shown to affect human cytomegalovirus (HCMV) with resulting IC_50_ of 2.6 and 3.7 μM, respectively [85].

Six briarane-type diterpenoids (briacavatolides A–F) and other two briaranes (briaexcavatolide U and briaexcavatin L) were recently isolated from the acetone extract of *Briareum excavatum* and examined for antiviral activity against HCMV using a human embryonic lung (HEL) cell line. Only Briacavatolides C and F demonstrated moderate anti-HCMV activity with IC_50_ of 18 μM and ED_50_ of 22 μM, respectively [103,104]. Four other compounds (sesquiterpenoids) isolated from *Paralemnalia thyrsoides* (Parathyrsoidins A–D), did not induce antiviral activity as observed in human embryonic lung (HEL) cells infected with HCMV [105].

Wang *et al.* [106] isolated three cembranoids, (+)-12-ethoxycarbonyl-11*Z*-sarcophine, ehrenbergol A, and ehrenbergol B from the acetone extracts of *Sarcophyton ehrenbergi*. All three compounds exhibited antiviral activity against HCMV with IC_50_s of 16.02, 13.20, and 1.37 μM, respectively. Acetone extracts from the same cnidarian yielded two other diterpenoids (ehrenbergol C and acetyl ehrenberoxide B) which showed activity against HCMV, with EC_50_ values of 52.84 and 21.95 μM, respectively [107].

Two steroids (echrebsteroids B and C) from the gorgonian *Echinogorgia rebekka* showed strong antiviral activity against respiratory syncytial virus (RSV) with IC_50_ of 0.19 μM. For another steroid (echrebsteroids A) IC_50_ value of 0.78 μM was reported [108].

Using a cell-based Chikungunya virus (CHIKV) replicon model, six norcembranoids and one new compound (kavaranolide) isolated using bioassay-guided chemical fractionation of the Indian soft coral *Sinularia kavarattiensis* were evaluated for their replicon-inhibiting potential. Moderate dose-dependent inhibition (more than 60% compared to the control) of CHIKV replicon was induced by norcembranoids 1 and 2 [109].

### 5.4. Antiparasitic Activity of Cnidarian Extracts

During late 1990s sea anemone venoms were tested for toxicity to the human intestinal parasite *Giardia duodenalis*. Anti-parasitic effects were shown for the cytolysins sticholysin I (St I) and II (St II) from *Stichodactyla helianthus* and for equinatoxin II from *Actinia equina* with IC_50_ values of approximately 10 ng/mL (St I) and 40 ng/mL (St II) [110]. A small amount (2%) of *Giardia duodenalis* was resistant to the sea anemone venoms. Subsequently, the activity of the extract from the jellyfish *Linuche unguiculata* against *Giardia lamblia* (IC_50_ = 63.2 μg/mL; 95% confidence interval: 63.3–63.1) was reported [102]. The same paper reports the antiprotozoal activity of extracts from other cnidarians against *Giardia lamblia*, but all IC_50_ values (*Cassiopea xamachana* = 226 μg/mL; *Bartholomea annulata* = 316 μg/mL; *Lebrunia danae* = 631 μg/mL; *Stichodactyla helianthus* = 1338 μg/mL) showed little effectiveness [102]. Ethanol extracts from *Muricea appressa* and *Pacifigorgia* sp. sampled from Baja California Sur (México) showed activity against *Giardia lamblia* and *Entamoeba histolytica*, with IC_50_ of 70 μg/mL and 72 μg/mL, respectively [82].

Cnidarian extracts were shown to be effective against microorganisms responsible for leishmaniasis (*Leishmania chagasi*) and Chagas disease (*Trypanosoma cruzi*). Methanolic crude extracts from the octocorals *Carijoa riisei*, *Heterogorgia uatumani* and *Leptogorgia punicea*, the hydroid leptomedusan *Macrorhynchia philippina*, the zoanthid *Palythoa caribaeorum*, the siphonophore *Physalia physalis*, and the hexacorals *Aiptasia pallida* and *Zoanthus sociatus*, as well as a modified steroid (18-acetoxipregna-1,4,20-trien-3-one) isolated from *Carijoa riisei* were tested. The extracts from *Carijoa riisei* and *Heterogorgia uatumani* were active, killing 50% of *L. chagasi* promastigotes at IC_50_ values of 2.84 and 4.40 μg/mL, respectively. All crude extracts were scarcely effective against *T. cruzi* (range of IC_50_ = 41.0–117.9 μg/mL). The modified steroid from *Carijoa riisei* showed antileishmanial effectiveness against promastigotes (IC_50_ = 5.5 μg/mL) and intracellular amastigotes (16.88 μg/mL) [111].

Recently Ishigami *et al.* [112] isolated the diterpenoid cristaxenicin A from the deep water gorgonian *Acanthoprimnoa cristata*. Despite its moderate antimalarial activity against *Plasmodium falciparum* (IC_50_ = 11 μM), cristaxenicin A showed effectiveness against *Leishmania amazonensis* and *Trypanosoma congolense* at IC_50_ of 0.088 and 0.25 μM, respectively. Furthermore, calyculone H isolated from *Eunicea* sp. showed activity against *P. falciparum* with IC_50_ value of 36.13 μM [83].

## 6. Conclusions

The discovery of antibiotics suggested that the battle between humans and microorganisms causing infections could be solved in a short time and a new era without infectious diseases had been entered.

However, the ability of microorganisms to respond to antibiotics with resistance mechanisms enormously complicated the situation. As a matter of fact, microbes have benefitted from overuse and misuse of antibiotics. Therefore, at present antibiotic/antimicrobial resistance and multi-resistance is the main problem to be faced to fight pathogenic microorganisms [23,113]. Significantly though, medical practices, such as cancer chemotherapy, organ transplantation, and all aspects of surgery would collapse without access to potent antibiotic therapy for prevention and treatment of infections [6,21].

Several events, such as the development of antibiotic resistant genotypes and the occurrence of other elements such as transposons, integrons, and insertion sequences, the development of resistance genes and gene transmission, have contributed to increased antibiotic resistance and to the development of multiple resistance mechanisms [23,114]. To date, genes mediating resistance are known to be spread by means of very efficient genetic mechanisms [6].

Several ways to face this challenge have been suggested, but new strategies to fight antibiotic resistance are necessary to develop new approaches to produce new substances and/or to strengthen the antimicrobial activity of currently used drugs [115]. Therefore, the development of new molecules or different therapeutic systems able to fight pathogenic viruses, bacteria, and fungi is imperative. Equally important is the need to find new natural sources of compounds with potent antimicrobial/antibiotic properties.

In this connection, considering that the oceans cover most of the Earth’s surface, marine organisms and their metabolites are thought to be a unique source of potential pharmaceutical substances—an enormous potential resource for the discovery of bioactive compounds. Among the substances that could be useful for this purpose are venoms and toxins, which seem to be particularly interesting for the development of new drugs. From this point of view, cnidarians are a research subject of pre-eminent interest, being included among the most venomous organisms inhabiting seawaters. At present, several interesting compounds have been isolated from cnidarians but, unfortunately, (with the exception of Pseudopterosin, Eleutherobin and some Sarcodyctins which have been evaluated in pre-clinical evaluations [63,64]) no compounds originating from cnidarian have to date been seriously considered for utilization. Essentially this appears to be due to the difficulties in sampling, the scarce availability of bioactive compounds, the small amounts of extracts and to the structural diversity of marine compounds [37]. This makes production by chemical synthesis or by using bred organisms desirable, but the costs of these procedures are thought to be not easily sustainable [45]. From this point of view, for example the frequent jellyfish outbreaks observed worldwide during the last decades [49] could have a positive application whereby a large amount of useful biologic material could be made available to transform a sanitary and economical problem into a useable resource.

At present, the therapeutic use of antibiotics is mandatory, therefore an adequate knowledge of the role of environmental microbiomes in the development of antibiotic resistance is needed [23]. The aim in acquiring such knowledge is to understand the role of antimicrobial compounds that are able to respond to environmental stimuli in the absence of conventional defense systems. The discovery of novel antibiotics and their fast introduction into anti-infective therapy is currently very desirable [23], therefore studies focused on gaining knowledge of structure and function of molecules synthesized by lower organisms, such as cnidarians, which are capable of producing antimicrobial compounds makes this research an interesting and important tool for the development of a novel generation of antibiotics [66].

Recently, details of the proteomes of jellyfish have emerged along with information on the molecular profile (using proteomic mass spectrometry approaches) of venom contained in nematocysts of dangerous jellyfish such as *Chironex fleckeri* [116]. It stands at present that characterisation of extracted constituents from cnidarian in general and their venom is the main obstacle in utilising the extracts therapeutically. This research on the cnidarian proteome is complicated by the presence of large proteins included in the venom, additionally the protein content can also be affected by the technique of preparation of venom for analysis [116,117]. For this reason, different extraction and fractionation methods can result in remarkable differences in results on the cnidarian proteosome [118]. Therefore, it is to be hoped that advances in proteomics studies will provide insight into jellyfish venomics with the outcome being the utilisation of therapeutically useful compounds.

In this connection, it is known that antimicrobial peptides (AMPs) play an important role in the innate immune system of marine invertebrates, which lack an acquired immune system [99]. In particular AMPs extracted from cnidarians, such as aurelin from *Aurelia aurita* [98] could have significant potential for the development novel antibiotics/antimicrobials [55,119]. The basis for this is that aurelin has shown good sequence and structural similarity to BgK and ShK, two potassium channel blockers isolated from sea anemones [99,116]. Aurelin also has partial similarity with defensins [98], antimicrobial molecules known to have a role in innate immunity in both invertebrates and vertebrates [120].

In conclusion, several cnidarian extracts as well as venom components are known to have interesting biological properties, therefore ongoing research investigating the use of cnidarians for development of new therapeutic antimicrobial drugs with potential to combat microbial resistance is highly attractive. Microbial resistance to antimicrobial therapeutics is regarded as one of the major medical issues confronting humans in the 21st century. Therefore cnidaria may open a new and exciting resource for the discovery of potent antiviral and antibacterial agents. In this connection economic resources and support from the pharmaceutical industry will be required to support the ongoing costs of a focused natural products research investigation on cnidarian. The future of potent antibiotics may very likely depend upon a unified and concentrated search for novel antimicrobial discoveries from the marine environment [106].

## Figures and Tables

**Table 1:** Activity of antimicrobials from freshwater cnidarians (Hydrozoa). For Hydramacin-1 and c-Arminin 1a the horizontal line separates Gram-negatives and Gram-positives. (1) two strains; (2) two strains of which one is multi-resistant; (3) three strains; (4) three strains of which two are multi-resistant; (5) three strains of which two are ESBL-producing; (6) four strains; (7) four multi-resistant strains; (8) four strains of which three are multi-resistant; (9) six strains of which five are MRSA; (10) eight strains of which five are multi-resistant; (*) three domains; (^) value in μg/mL. MBC = Minimum Bactericidal Concentration; LD = Lethal Dose; ESBL = Extended-Spectrum Beta-Lactamase; VRE = Vancomycin-Resistant Enterococci.

Species	Compound	Sensitive Microbes	Activity		Reference
			MBC (μM)	LD_90_ (μM)	
*Hydra magnipapillata*	Hydramacin-1 (peptide)	*Citrobacter freundii*	0.9	0.2	[66]
		*Citrobacter freundii*	4.0 ± 4.4	0.7 ± 0.3	[67]
		*Enterobacter cloacae*	1.8	0.4	[66]
		*Enterobacter cloacae* ^(1)^	7.6 ± 9.5	0.7 ± 0.3	[67]
		*Escherichia coli* ^(10)^	1.5 ± 2.3	0.3 ± 0.1	[66]
		*Escherichia coli* ^(2)^	2.25 ± 1.9	0.55 ± 0.5	[67]
		*Salmonella typhimurium*	0.9	0.2	[66]
		*Salmonella typhimurium* ^(1)^	0.9 ± 0	0.5 ± 0	[67]
		*Yersinia enterocolitica*	0.4	0.2	[66]
		*Yersinia enterocolitica*	0.9	0.2	[67]
		*Klebsiella oxytoca* ^(7)^	0.9 ± 0	0.35 ± 0.1	[66]
		*Klebsiella oxytoca* ^(4)^	3.9 ± 3.1	0.8 ± 0.2	[67]
		*Klebsiella pneumoniae* ^(8)^	1.8 ± 1.3	0.5 ± 0.25	[66]
		*Klebsiella pneumoniae* ^(2)^	2.25 ± 1.9	0.7 ± 0.3	[67]
		*Acinetobacter baumannii*	7.1	1.8	[67]
		*Proteus mirabilis*	14.3	0.9	[67]
		*Proteus vulgaris*	>14.3	3.6	[67]
		*Pseudomonas aeruginosa*	>14.3	>14.3	[66]
		*Serratia marcescens* ^(3)^	>14.3	6.6 ± 6.7	[67]
		*Bacillus megaterium*	0.2	0.1	[66]
		*Enterococcus faecalis*	14.3	0.9	[66]
		*Enterococcus faecalis* ^(10)^	>14.3	7.1	[67]
		*Staphylococcus aureus*	>14.3	14.3	[66]
		*Staphylococcus hominis*	>14.3	3.6	[66]
		*Staphylococcus hemolyticus*	1.8	0.9	[67]
		*Streptococcus pyogenes*	>14.3	7.1	[67]
	Periculin-1 (recombinant peptide)	*Bacillus megaterium*	Not given	0.2–0.4	[66]
	c-Arminin 1a (recombinant peptide)	ESBL-producing *K. pneumoniae* ^(3)^	0.5 ± 0.2	0.2 ± 0	[68]
		*Escherichia coli* ^(5)^	0.3 ± 0.1	0.08 ± 0.03	[68]
		*Bacillus megaterium*	0.1	0.01	[68]
		*Staphylococcus aureus* ^(9)^	0.5 ± 0.2	0.17 ± 0.06	[68]
		*Enterococcus faecalis* (VRE)	1.6	0.8	[68]
		*Enterococcus faecium* (VRE) ^(6)^	0.5 ± 0.2	0.2 ± 0	[68]
	Kazal-2 (native protein)	*Staphylococcus aureus*	0.7–0.8	-	[69]
	Kazal-2 (recombinant protein) (*)	*Staphylococcus aureus*	35.7 ± 2.5	-	[69]
*Hydra* spp.	Arminin (peptide)	*Escherichia coli*	1.823 ± 1.19 (^)		[70]

**Table 2:** Activity of antimicrobials from marine Anthozoa and Scyphozoa (^). Results of MIC values. (*) = Microplate Alamar blue assay. MIC = Minimum Inhibitory Concentration.

Species	Compound	Sensitive Microbes	MIC (μg/mL)	Reference
*Pseudopterogorgia elisabethae*	Pseudopteroxazole (diterpenoid alkaloid)	*Mycobacterium tuberculosis* H37Rv	15	[73,75]
	Pseudopteroxazole	*Mycobacterium tuberculosis* H37Rv	15 ^(^*^)^	[75]
	(Ptx-CH_2_-(1H-imidazol-5-yl))	*Mycobacterium tuberculosis* H37Rv	13 ^(^*^)^	[75]
		*Mycobacterium smegmatis*	4	
		*Mycobacterium diernhoferi*	2	
	iso-Ptx-H	*Mycobacterium tuberculosis* H37Rv	14 ^(^*^)^	[75]
	Ptx-CH_3_	*Mycobacterium tuberculosis* H37Rv	15 ^(^*^)^	[75]
	Pseudopterosin P	*Streptococcus pyogenes*	0.8	[77]
		*Staphylococcus aureus*	2.0	
		*Enterococcus faecalis*	3.5	
	Pseudopterosin Q	*Streptococcus pyogenes*	1.0	[77]
		*Staphylococcus aureus*	2.3	
		*Enterococcus faecalis*	3.6	
	Homopseudopteroxazole (diterpene alkaloid)	*Mycobacterium tuberculosis* H37Rv	12.5	[74]
*Nephthea* sp.	Litosterol	*Mycobacterium tuberculosis*	3.13	[83]
	Nephalsterol C	*Mycobacterium tuberculosis*	12.5	[83]
*Sarcophyton glaucum*	Sarcophytolide (lactone cembrane diterpene)	*Staphylococcus aureus*	0.19	[88]
		*Pseudomonas aeruginosa*	0.22	
		*Saccharomyces cerevisiae*	0.13	
*Sarcophyton trocheliophorum*	Cembranolide	*Staphylococcus aureus*	125	[89]
*Xenia macrospiculata*	Desoxyhavannahine	Marine bacteria	48	[90]
*Aurelia aurita* (^)	Aurelin (peptide)	*Listeria monocytogenes*	5.27	[98,99]
		*Escherichia coli*	1.78	
		*Bacillus megaterium*	10	
		*Micrococcus luteus*	40	
